# Assessing the socio-economic burden of inherited and inflammatory neuromuscular diseases (BIND study): a study protocol

**DOI:** 10.1186/s13023-025-03904-z

**Published:** 2025-08-06

**Authors:** Ian C. Smith, Yasmin Abusetah, Homira Osman, Aditi Garg, Alyssa Grant, Hanns Lochmuller, Hugh McMillan, Gerald Pfeffer, Lawrence Korngut, Cynthia Gagnon, Stacey Lintern, Daria Wojtal, Kathy Selby, Kednapa Thavorn, Jodi Warman-Chardon

**Affiliations:** 1 The Ottawa Hospital Research Institute, Ottawa, ON Canada; 2 Faculty of Science, University of Ottawa, Ottawa, ON Canada; 3 Muscular Dystrophy Canada, Toronto, ON Canada; 4 University of Ottawa Heart Institute, Ottawa, ON Canada; 5 Faculty of Medicine, University of Ottawa, Ottawa, ON Canada; 6 Department of Medicine (Neurology), The Ottawa Hospital/ University of Ottawa, Ottawa, ON Canada; 7 Children’s Hospital of Eastern Ontario, Ottawa, ON Canada; 8 Department of Clinical Neurosciences, Cumming School of Medicine, Hotchkiss Brain Institute, University of Calgary, Calgary, AB Canada; 9 Department of Medical Genetics, Cumming School of Medicine, Alberta Child Health Research Institute, University of Calgary, Calgary, AB Canada; 10 University of Sherbrooke, Sherbrooke, QC Canada; 11 British Columbia Children’s Hospital, Vancouver, BC Canada; 12 School of Epidemiology and Public Health, University of Ottawa, Ottawa, ON Canada

**Keywords:** Neuromuscular disease, Socio-economic burden, Cross-sectional survey

## Abstract

**Introduction:**

Neuromuscular diseases (NMDs) are rare multisystem, genetic or acquired disorders causing weakness and/or sensory loss. It is essential for governments, insurance providers, and broader society to have a better understanding of the burden of illness of NMDs. Our goal is to assess the social and economic burden of Canadians living with NMDs, encompassing schooling and education achievement, health-related quality-of-life, and labour force participation and productivity.

**Methods and analysis:**

We will conduct a national, cross-sectional survey of individuals living with a NMD and their caregivers who are members of Muscular Dystrophy Canada and/or are patients within our national network of neuromuscular clinics. Surveys can be completed online or via telephone. The specific sub-sections of the questionnaire will differ based on respondent’s profile, whether they are 1) a minor living with a NMD, 2) an adult living with a NMD, 3) an adult who is a caregiver for someone living with a NMD, or 4) an adult who both lives with a NMD and is a caregiver for someone with a NMD. We will use descriptive statistics to describe distributions and ranges of the social and economic measures. Pearson correlations for continuous data and Spearman rho for rank data will be used to detect the strength of association of socio-demographic factors, disease characteristics, and social and economic impacts of NMDs.

**Ethics and dissemination:**

The study protocol has been approved by the Ottawa Health Science Network Research Ethics Board (Protocol ID # 20210601-01H). This study will provide the overall impact of NMD on costs and health-related quality of life, disseminated via a series of manuscripts which will include both between- and within-NMD/NMD subtype comparisons. The data obtained will guide governmental policy development and inform patient organisation programs to deliver more effective supports to individuals and families affected by NMDs.

## Introduction

Although individually rare, collectively, the 600 genetic and acquired neuromuscular disease (NMD) subtypes affect an estimated 1 person per 500 [1], equivalent to 76,000 Canadians. Many NMDs are characterized by profound weakness and/or sensory loss and have multisystem involvement including cardiac and respiratory failure or intellectual delay [2, 3]. Importantly, new disease-modifying therapies are emerging from clinical trials and persons with genetic and acquired NMDs are receiving more complex care at home [4, 5]. In Canada, approximately 70% of direct health care expenditures, including physician services, diagnostic tests, and hospitalization expenses, are covered by government funding [5, 6]. The remaining 30% of direct health care expenses are either covered by private health plans or become out-of-pocket expense for patients [5]. The direct costs of NMDs, including medical services, personal support workers, home rehabilitation services, travel, lodging, home renovations (*e.g.*, ramps) and wheelchair accessible vehicles pose considerable socio-economic burdens to individuals and their caregivers. The indirect costs associated with NMDs, including lost production due to morbidity, premature mortality, and informal caregiving can also be considerable [6–8].

From the perspective of patients and caregivers, the actual out‐of‐pocket and indirect expenses are most critical, but they are largely “invisible” in most economic evaluations of NMD treatments. The indirect costs of many NMDs in Canada have not been assessed comprehensively, with existing data being limited to single centres or single diseases [5, 9–11]. Furthermore, fragmented healthcare and social security systems within Canada [12, 13] contribute to the difficulties studying the economic burden of rare diseases. Results obtained from other countries may not be generalizable to Canada due to differences in public and social policy and payment mechanisms [14]. An improved understanding of the burden of NMDs is essential for governmental agencies, insurance providers, patient partners, and broader society deliver effective supports to those individuals with NMD and their caregivers.

**Fig. 1:**
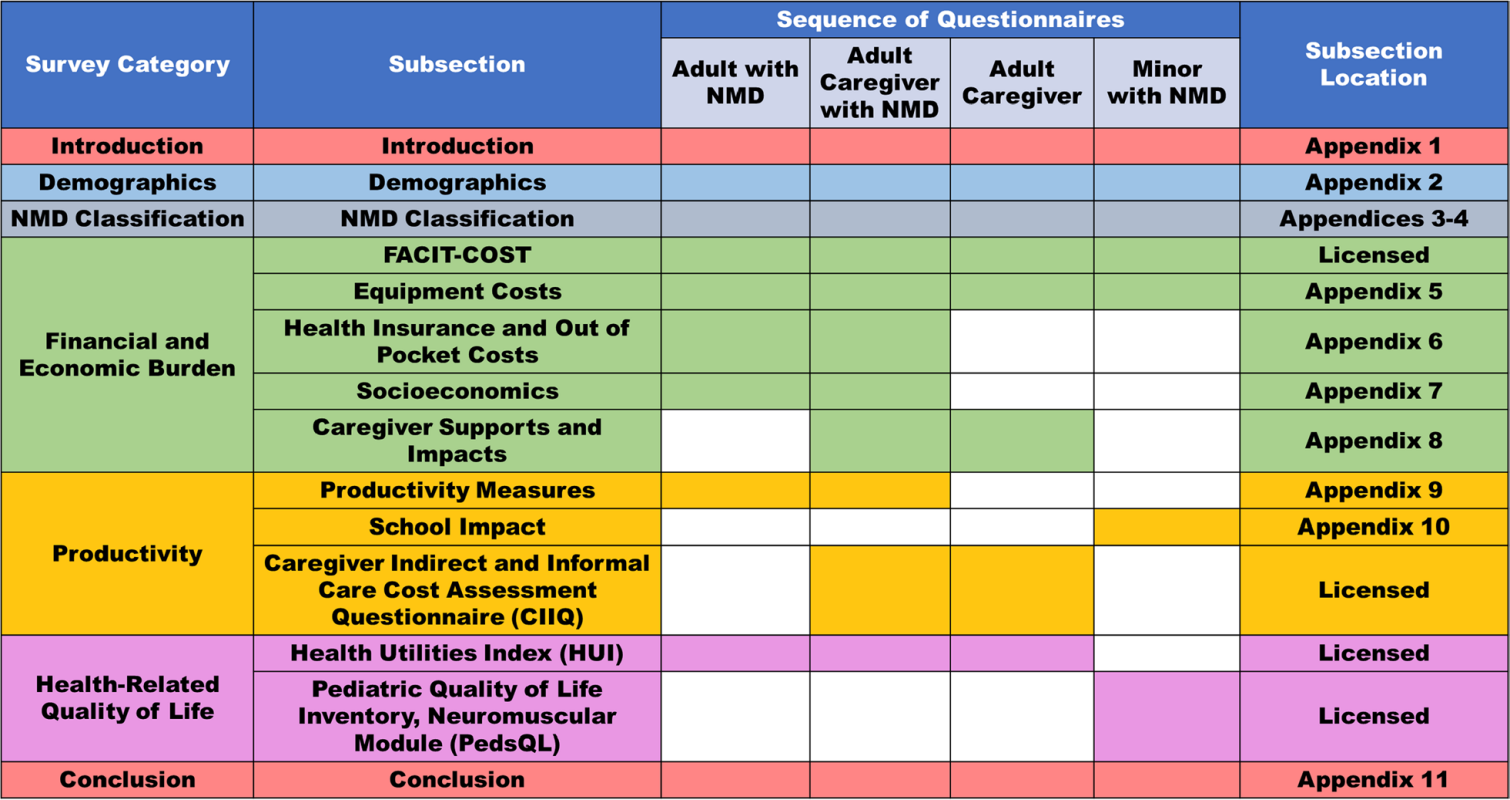
Survey framework. The sequence of included subsections for each participant category is shown with shaded cells. Subsections can be found in the appendices of this manuscript, or licensed from the copyright holder

This study aims to measure both the financial and broader social impact of NMD on patients and their caregivers. It will explore various, including educational attainment, health-related quality of life (HRQoL), and labour force participation and productivity (Fig. 1). Our research will inform governmental policy development and inform patient organisation programs to better support individuals with NMDs and their families.

## Methods

We will conduct a national, cross-sectional survey of individuals with a NMD, and family caregivers experienced with NMDs (including Duchenne muscular dystrophy, myotonic dystrophy, Charcot-Marie-Tooth disease, limb girdle muscular dystrophies, among others). This study design was a collaboration between NMD clinicians, patient partner organizations, and health economists. The survey will be completed online, or with assistance of a Muscular Dystrophy Canada (MDC) study representative via telephone. The informed consent form will be accessible on the study landing page website, and informed consent will be implied by the completion of the questionnaire. All study materials will be available in French and in English.

### Sample size estimation

An internal survey conducted over 7 months in 2021 by Muscular Dystrophy Canada received over 600 responses from individuals with six relatively common NMDs: Duchenne muscular dystrophy, myotonic dystrophy, facioscapulohumeral muscular dystrophy, Charcot-Marie-Tooth disease, myasthenia gravis, and spinal muscular atrophy. As BIND covers a broader range of NMDs, has additional visibility and support through the Neuromuscular Network of Canada and the Canadian Neuromuscular Disease Registry, and will be open for a longer duration, we anticipate receiving over 1,000 responses. NMDs with prevalence of 3.0 to 40.0 per 100,000 (total populations of 1,200–16,000 in Canada) such as myotonic dystrophy [15], myasthenia gravis [16], Charcot-Marie-Tooth disease [17], spinal muscular atrophy [18], facioscapulohumeral muscular dystrophy [19], and oculopharyngeal muscular dystrophy [20] will require 90–96 participants to achieve a 10% margin of error with a confidence interval of 95%. NMDs with prevalence of 1–3 per 100,000 (total population of 400–1,200 in Canada) such as limb girdle muscular dystrophy [19], Becker muscular dystrophy, and Duchenne muscular dystrophy [21] will require sample sizes of 78–90 to achieve a 10% margin of error with a confidence interval of 95%. NMDs with prevalence between 0.1 and 1.0 per 100,000 (total population of 40–400 in Canada) such as autosomal recessive spastic ataxia of Charlevoix-Saguenay [22], multifocal motor neuropathy, Emery-Dreifuss dystrophy, Lambert-Eaton myasthenic syndrome [23], Pompe disease [24], and spinobulbar muscular atrophy [25] will require sample sizes between 29 and 78 to achieve a 10% margin of error with a confidence interval of 95%. Respondents will be asked to be as specific as possible with their diagnosis to enrich the types of comparisons that can be conducted within a particular type or family of NMDs. As many NMDs are extremely rare, comparisons between different NMDs will be facilitated by generating subgroups of NMDs determined based on the number of responses received and clinical/causative features of the reported NMD. NMD subgroupings will be conducted by a medical practitioner specializing in rare NMDs, with possible examples of terms including autoimmune myopathies, congenital myopathies, ataxias, myotonic dystrophies, as well as an “other NMDs” category to enable inclusion of extraordinarily uncommon diagnoses and individuals with an NMD that has not been definitively diagnosed.

As the progressive nature of many NMDs can cause substantial variability in the clinical presentation even amongst individuals with the same diagnosis, financial burden of NMD may be more dependent on specific disease features (*e.g.,* difficulty with ambulation) than on a specific diagnosis. Therefore, we plan to perform analyses using extra-diagnostic participant characteristics provided in responses to PedsQL and/or HUI questionnaires (*e.g.,* individuals who [can/cannot] walk independently, and those who [do/do not] have vision/hearing/speech/communication impairment due to their NMD). We also plan to perform comparisons of caregivers based on CIIQ responses, for example, care givers who [have/ have not] had to give up paid employment due to the demands of caregiving. This approach will increase the robustness of the conclusions that can be drawn and will also better accommodate the NMDSs that cannot be analyzed in isolation due to their rarity.

### Recruitment strategy

Individuals with NMD and their caregivers who are registered with MDC (www.muscle.ca) will be invited to participate in this study. Participants will be invited via email from the membership lists of MDC, as well as via the Canadian Neuromuscular Disease Registry (CNDR; [4]). Advertisements will be shared via MDC’s website, social media platforms, email newsletters, and seminars. Patients registered with MDC typically receive newsletters with information specific to their NMD. Advertisements for BIND may be included in these newsletters to help raise awareness in the specific subpopulations to encourage representative sampling. Like-minded NMD-focused patient partner organizations (*e.g.*, CureSMA, www.curesma.org; Defeat Duchenne Canada, defeatduchenne.ca) will be provided with electronic copies of the study poster to circulate to their members. We will also recruit individuals with NMDs from over 20 neuromuscular clinics across Canada through the Neuromuscular Network for Canada (NMD4C; Neuromuscularnetwork.ca) clinician-scientist NMD network. NMD Neurology and Physiatry clinics interested in publicizing the study will be given posters to alert patients to the study. With a large clinical presence and additional visibility from the NMD4C, CNDR etc. to promote the BIND study, we anticipate responses from over 1,000 individuals living with NMD.

### Target population

Our study will focus on the following populations:

1. Adults aged 18 years or older who are living with NMD
2. Children living with a NMD (2–17 years old)
3. Adult caregivers (without a NMD) of those affected by NMD
4. Adult caregivers who are living with a NMD and caring for others affected by it

Each survey questionnaire is intended to correspond to a single individual. At the household level, ideally, one survey would be completed per person living with a NMD, and one survey would be completed per caregiver. As part of the implementation, surveys from the same members of a household will be linked together using a “household code” generated for respondents who indicate there is another member of the household who would like to complete the survey.

### Inclusion criteria

Survey respondents must reside in Canada, be fluent in English or French and self-report with one or more clinically diagnosed neuromuscular condition (Table 1). Caregivers are eligible if the are ≥ 18 years old, caring for an individual with a NMD and can read or communicate in English or French. A caregiver is defined as someone with a personal relationship, such as a parent, spouse, partner, or adult relative who provides unpaid care for an individual with NMD [5] Parents or guardians will complete surveys on behalf of minors with NMD.

### Exclusion criteria

Individuals with NMD less than 2 years of age, individuals without a NMD diagnosis (excepting caregivers), individuals not living in Canada and formal/paid caregivers for individuals with a NMD are ineligible to participate in the study.

### Identifying information collected

Participants will be given the option to complete the survey anonymously, or with identifiable information (name and email) visible to MDC who can aid with survey completion and verification of NMD diagnosis based on their registry data.

### Pause and resume functionality

While all progress will be automatically saved, respondents will be given the ability to pause and resume their survey in progress with an option to schedule assistance from a representative of MDC. The ability to resume a survey in progress requires respondents to request a secure link to be emailed to them via an automated process. As such, the ability to resume a survey in progress will not be available to individuals wishing to remain completely anonymous.

### Incentives

No incentives will be offered for initiating or completing the survey. While offering incentives for survey completion would motivate a larger proportion of the target population individuals to complete the survey [26], it would also incentivize low quality or fraudulent survey responses which may not be easy to filter from a data integrity perspective [27].

### Online implementation and hosting

The online survey will be implemented and securely hosted using proprietary software of the Ottawa Methods Centre at The Ottawa Hospital. Data will be accessible to designated research staff through an online, password-protected, access portal. It is anticipated that the survey will be active for recruitment for a period of 12 months, after which, the database will be archived for analysis.

#### Measurement and survey questionnaires

Guided and informed by patients and parent research partners, Fig. 1 depicts the different questionnaires that will be used for caregivers, adult participants with NMDs, and on behalf of minors with NMDs. Depending on the respondent’s profile, the appropriate set of questionnaires will be launched. As all responses are voluntary, advancement through the online survey is not encumbered by the requirement for a response. Representatives from MDC were involved in all aspects of the survey design, including study conception, design of the general framework, inclusion/exclusion/wording of specific questions, and refinement based on feedback received during pilot testing. Pilot testing consisted of a panel of 15 eligible participants independently completing the online survey to ensure that the questions were clearly articulated, and the response options are relevant and comprehensive from the point of respondents. The panel of eligible participants consisted of 5 parents/guardians of children with NMD, 5 adults with NMD, and 5 caregivers for someone with NMD. Based on participant feedback, the survey was expanded to include questions regarding expenditures on home and vehicle modifications, and durable medical equipment needed for the NMD. Based on pilot testing, we anticipate that the combined online survey completion time will be 30–45 min.

#### Questions for all participants

We will collect socio-demographic data, including month and year of birth, sex, gender, ethnicity, language primarily spoken at home, and first 3 characters in their postal code. Disease characteristics, including diagnosis and age at diagnosis, will be collected for all participants living with NMD. Participants will be asked how many members of their household are afflicted by NMD. At the completion of the survey, participants with multiple affected members in the same household will be linked by a household identification number.

To gauge the financial distress experienced by individuals with NMD and their caregivers, participants will be asked to complete the FACIT – COST measure of financial toxicity [28]. The FACIT – COST requires respondents to rate 11 statements relating to financial toxicity on a five-point Likert scale. Lower scores represent worse financial toxicity. The FACIT – COST measure was originally developed for cancer patients [28], but has been used with individuals with chronic diseases such as diabetes [29, 30]. All participants will also be asked to provide detailed cost breakdowns for home and vehicle modifications and mobility/assistive devices needed due to NMD encountered in the past 5 years. Though a 5-year timeframe may have increased risk of recall bias, this timeframe was chosen based on feedback from our patient-partners test panel who felt it was necessary to capture the large but infrequent expenditures which have a substantial impact on household finances and on quality of life.

#### Questions for all adults

The economic burden is conceptualized as psychosocial as well as direct and indirect costs. In this study, we will focus on the impact of NMDs on schooling, education attainment, labour force participation, quality-adjusted life expectancy (QALE), and indirect costs (lost earnings and productivity by the patient or caregivers attributed to NMD diagnosis). Study questionnaires are based on the existing validated and standardized questionnaires to measure the disease burden. We will inquire about their marital status, employment status, educational attainment, occupation, and income level to assess the living situation of adult individuals with NMD and caregivers.

We will use a validated and standardized questionnaire, the Health Utilities Index (HUI®) to describe health status and to obtain utility scores of multi-attribute health-status classification systems [31]. The HUI assesses eight attributes of quality of life: vision, hearing, speech, ambulation, dexterity, emotion, cognition, and pain; it has been demonstrated to provide more discrimination across functional status most relevant to genetic NMDs [32]. HUI scores will be calculated using the HUI3 health status classification system, with the score ranging from − 0.36 (worst possible health state) through 0.00 to 1.00 (perfect health) [31]. HUI is a well-validated instrument and has been used to assess HRQoL of individuals with NMDs and their caregivers [9, 10, 32] as well as the Canadian household population aged 5 years or older. It has demonstrated good discriminant validity and high test–retest reliability (intraclass correlation coefficient of 0.77) [33]. The estimated utility values will be used to estimate QALE.

#### Questions for adults with NMD only

Adults with NMD will be asked about their level of schooling, work/employment status, and personal and household income. Jobs will be classified by the system used by Statistics Canada. As with the Statistics Canada’s Labour Force Survey, we define labour force participation as having a paid job at the time of the study. This measure allows for comparison of results to the full Canadian population as well as a group of Canadians affected by neurological conditions. Adults with NMD will be asked about their access to private health insurance, medications used to manage their NMD, and out-of-pocket costs concerning the care and management of their NMD. Adults with NMDs will be asked about absenteeism, presenteeism, and impairments in unpaid activity because of NMDs using a productivity measures questionnaire. The productivity measures questionnaire is an adaptation of the Work Productivity and Activity Impairment questionnaire (WPAI) [34], which is used to measure the impairments faced at work and amount of missed from work (paid and unpaid work) due to health problems [34–36]. Based on the recommendations of our patient partners and on published work [32], the WPAI was altered to include productivity while at school and how the type and quality of work performed may be affected by a NMD.

#### Questions for caregivers only

Adults indicating that that they are informal caregivers for someone with a NMD will be asked for the diagnosis/diagnoses and age(s) at diagnosis for all associated individuals affected by NMD. To assess the economic cost to caregivers, we will use the Caregiver Indirect and Informal Care Cost Assessment Questionnaire (CIIQ) to measure, value, and estimate caregiver indirect (productivity) and informal care costs [37]. The questionnaire contains 13 questions regarding caregiver current and previous work status, productivity, and the provision of informal care [37]. CIIQ assesses data of cost components, regardless of the patient’s disease, condition, or location [37]. Caregivers will also be asked if they had to relocate as a result of care the needs of the person(s) affected by NMD.

#### Questions for minors with NMD only

Parents or guardians completing surveys on behalf of minors with a NMD will be asked to indicate if the minor in their care is currently attending school, the highest level of schooling achieved, school missed in the past 7 days and in the entire life of the minor with NMD. HRQoL will be probed using the PedsQL Neuromuscular Module Version 3.0 Parent report for Child [38, 39]. Implemented through a Likert 5-point scale, this assessment enables both children and their caregivers to evaluate the impact of the disease on various aspects of the child’s life over the preceding month. The PedsQL 3.0 Neuromuscular Module underwent rigorous validation through quantitative testing, ensuring its reliability in measuring vital quality of life constructs relevant to pediatric individuals living with NMD [38, 39].

## Data analysis

### Social and economic burden of individuals with NMD

#### Patients and caregivers experience with NMDs

We will use descriptive statistics to calculate distributions and ranges of the social and economic measures. Pearson correlations for continuous data and Spearman rho for rank data will be used to detect the strength of association of socio-demographic factors, disease characteristics and social and economic impacts of NMDs.

Labour force participation of individuals with NMDs will be stratified for type of disease, age, sex, gender, and educational level. We will adjust the age-sex-educational level distribution in the study group to the distribution in the general Canadian population by using the direct standardization method and compare the standardized labour force participation rates with those reported by Statistics Canada [40]*.* Based on the human capital approach, we will calculate the indirect costs of NMDs by multiplying productivity loss (absenteeism) obtained from the productivity measures questionnaire (adults with NMD) or the CIIQ (caregivers) questionnaire with the average expected salary for a person in 2023 in the same age group, sex, and occupation type [41]. The total indirect costs will represent lost production to due morbidity associated with NMDs and be equal to the sum of lost earnings and productivity by the patients and caregivers experienced with NMD. As a scenario analysis, we will also calculate the indirect cost using the friction cost method to allow comparison of our estimates with other studies [42]. Additionally, we will calculate QALE by multiplying the number of years that an individual can expect to live, *i.e.*, life expectancy [43], with health utility values derived from the HUI.

To avoid duplication of costs (*e.g.*, the cost of a wheelchair being reported by an adult with NMD and by their caregiver), data from households with multiple respondents will be assessed and combined when needed. Complementary to the software-coded method of linking household members, manual identification of households is possible by identifying responses with a high degree of overlap on responses such as forward sorting address, the number of affected individuals, the specific diagnosis/diagnoses, household income, language spoken at home, ancestry, and date and time of completion. Duplicated responses for a single individual can be distinguished from different entries from a single household by examining month and year of birth along with the number of affected individuals to rule out occurrences of multiple affected births. Respondents providing contact information can also be contacted by MDC to obtain clarification. Additionally, if respondents specifically mention that another household member has already responded and provide sufficient details to identify that response (*e.g.,* by time and date of survey completion, age, common forward sorting address, specific diagnosis, etc.), a manual linkage can be created by research staff. Where duplicate/divergent responses within a household exist, and clarification cannot be obtained, the response used will be that of the most complete questionnaire, or if both are complete, the most recently completed questionnaire will be used. Specific procedures for combining data for members of a single household will be described in subsequent publications reporting results of the BIND study. Responses which are not linked will be considered independent households. The possibility that some responses from different members of a household may not be linked is a limitation of the study.

#### Impact of disease characteristic and management on social and economic burden

Multiple regression analyses will be used to investigate the relationship between each aspect of social and economic burden of NMDs and demographics, disease-related characteristics, and disease management in a series of manuscripts reporting results from the BIND study. This approach will permit analyses on the impact of heterogeneities between different NMD subtypes, and severity of presentation within an NMD (*e.g.,* by HUI or PedsQL score) on social and economic burden. Differences in the availability and accessibility of effective treatments will be reflected in these analyses as appropriate to the condition(s). The model performance, including goodness of fit and specifications, will be examined by checking the scaled deviance, Pearson’s χ^2^ statistics and residual plots, respectively.

#### Sex and gender-based analysis (SGBA)

Sex and gender are known determinants of access to care, the burden of disease and caregiving burden [44–46]. SGBA is also an important consideration for this study given known sex differences in the prevalence and severity of many NMDs [47–52]. Some genetic neuromuscular diseases, (*e.g.*, Duchenne muscular dystrophy and spinal and bulbar muscular atrophy) are sex chromosome-linked which result in male-exclusive disorders, and milder phenotypic variations of these conditions in a subset of female carriers [53, 54]. We will explore the data for sex and gender differences in schooling and education achievement, HRQoL, labour force participation, work productivity and indirect costs. We will also attend to the nature of our recruitment to ensure equitable access to entry into the study, including posting recruitment notices in different areas of the NMD clinics (*e.g.*, entrance, waiting room) and on social media pages. Sampling and analysis will ensure that we obtain diverse gender perspectives. Knowledge translation strategies will also incorporate sex and gender lenses [55].

#### Fraudulent, duplicate, and incomplete responses

As no incentives are offered for survey initiation or completion, we anticipate receiving few fraudulent entries. Moreover, a large proportion of participants are anticipated to be registered with MDC and have the option to include their name and email, resulting in a large subset of the respondents having verifiable responses. Survey responses without a verifiable identity can be assessed for evidence of response inattention or fraudulent activity using strategies described elsewhere [27]. As several publications are expected to come from this study, the treatment of incomplete surveys and missing data will depend on the specific research question(s) being assessed. In general, a subset of necessary responses will be identified for a particular research question, and survey responses which are incompatible with analysis will be excluded and reported as such with specific rationale for exclusion.

## Discussion

This project will assemble and assess one the largest cohorts of individuals with NMDs in Canada. By assembling a large cohort, we can estimate indirect social and economic burden, as well as its determinants among individuals with NMD and caregivers for individuals with NMD. The indirect cost estimates can be used to support future cost-effectiveness analyses of novel therapies for genetic and acquired NMDs. This evidence will be increasingly important as public and private payers will seek ways to rationalize the total economic burden given the increasing health care expenditures and the high cost of newer NMD therapies [56–61].

Our direct link with the MDC and other NMD communities will allow us to identify individuals with NMD and their caregivers experiencing the highest economic and social burden. Though individuals with NMD can self-refer for the study, a large proportion of respondents will have verifiable NMDs through their registration with MDC, which requires diagnosis to be confirmed by a medical specialist. Given the large number of individuals registered with MDC, we will perform sub-group analysis where possible to identify disease-specific differences in financial and economic burden. Given sex and gender are important determinants of access to care, the financial burden of disease and caregiving burden, we will explore the data for sex and gender differences including HRQoL, labour force participation, work productivity, and indirect costs in schooling and education achievement. Individuals with NMD often experience fatigue [62] and by offering telephone assistance and the ability to pause, save, and resume survey progress, the survey will be more accessible to more individuals with NMD or their caregivers.

This study will also capture the indirect and direct burden of NMD in Canada, allowing for the first time, cross-jurisdictional and regional comparisons within jurisdiction. Therefore, our research will inform health system planning based on disease-specific and region-specific data (*e.g.*, urban vs rural). Important practical applications of our research include better identification of individuals with NMD and their caregivers most in need of social and economic supports. This study will enhance the ability of patient organizations and government to make informed decisions to best support the incurring the highest direct and indirect burden, by influencing policy in health, vocational, community and school settings. A unique feature of this study is its inclusiveness to the breadth of NMDs. By this approach, individuals with rare and under-assessed diseases can have their voices heard alongside those with more common NMDs [63]. The inclusive approach of this study may be adaptable to the study of other families of rare disease.

### Anticipated findings

Based on previous studies, we hypothesize that NMDs with a higher prevalence/severity of disability, reduced availability of treatment, and/or requiring long-term resource intensive management will be associated with reduced labour force participation, health utilities and quality adjusted life expectancy and increased financial toxicity, direct and indirect costs among patients and caregivers [14, 64–66]. The types of occupations held may also vary by age of symptom onset and severity of disability [67], for example those who enter the workforce after the onset of their illness may be more likely to work in an office setting [68]. Further, factors including age, sex, and ethnicity may influence the associations of NMD types with the outcomes of interest [66, 69, 70].

There are several potential limitations of this study. We cannot capture experiences of individuals not fluent in French or English, which could reduce the response rate of specific populations (*e.g.*, newly immigrated Canadians) in Canada. Self-referral survey captures highly-motivated and engaged individuals [71]. Those with busy lifestyles may be less-likely to engage [72]. As the survey is designed to be completed once per person, large families will have a larger time cost associated with survey completion; this is an important consideration for hereditary conditions in which multiple family members could be affected. There is an additional burden for caregivers living with a NMD to fill out the study, as they are filling this out as an adult/caregiver with NMD as well as on behalf of their affected child or parent. This study requires phone or internet access; advertisements are almost exclusively online. Individuals with no or limited internet access would be less likely to be aware of the study and be less likely to participate in the study. For example, populations such as Mennonites, Hutterites, rural Canadians, some Indigenous communities, and people from underprivileged socioeconomic backgrounds could be underrepresented. As many individuals with NMD are followed at less-than-yearly intervals, advertisements in clinics will not be visible by some proportion of individuals with NMD in the time frame of the study. To help mitigate underrepresentation of individuals with physical, learning, or cognitive difficulties which would limit participation, caregivers are asked to submit responses on behalf of loved ones who wish to participate but lack the means to fully participate on their own.

## Conclusion

The estimates of social and economic burden among individuals with NMD and caregivers has not been well established for many NMD. By identifying the known as well as hidden financial costs, this study will provide data for future, improved cost-effectiveness analyses regarding treatment planning. Also, the results of this study will better inform the health system, by providing necessary data for patient organizations and governments to make informed decisions, influencing policy in health and support financially vulnerable individuals. Importantly, the strong involvement of MDC and patient partners ensures that this research remains focused on individuals with NMD.

## Data Availability

The questionnaires described in this study are either provided in the supplementary information or are available to be licensed from the copyright holders described within this manuscript. No datasets are included in this protocol paper.

## Appendix 1: survey introduction (all participants)

**BIND study: assessing the indirect socio-economic burden of inherited neuromuscular diseases**

This survey will assess costs associated with living with a neuromuscular disease (NMD) or caring for someone with an NMD and the impacts on quality of life. The purpose of this survey is to better understand the social and cost impact of NMDs on patients and their caregivers in Canada.

This is a research project being conducted by the research team at the Ottawa Hospital Research Institute (OHRI) in collaboration with partner organizations including Muscular Dystrophy Canada (MDC), the Neuromuscular Disease Network for Canada (NMD4C), and the Canadian Neuromuscular Disease Registry (CNDR).

Participation is voluntary. Your responses are anonymous. Even though the likelihood that someone may identify you from the study data is very small, it can never be eliminated.

The participant informed consent form provides more details about the study.

By completing this survey, you are providing your consent for members of the study team to view and use your responses and comments as data in this research study.

You can email *[Muscular Dystrophy Canada email address]* or call *[Muscular Dystrophy Canada Phone Number]* if you have questions or would like to schedule a time to complete the questionnaire by phone.

It will take you approximately 40 min to complete the surveys.

[Click here to start the survey.]

## Appendix 2: demographics questionnaire (all participants)

1. I am aged 18 years or above. Yes/No.

2. Please Enter your name.

3. Please Enter your email address.

I am completing this survey…

A: …as (or on behalf of) a person with a NMD who is >18 years old.

B: …as a person with a NMD who is > 18 years old. I am also a caregiver who provides routine care for a child/partner/relative/friend with a NMD.

C: …on behalf of a child or teenager (< 18 years old) who has a NMD. I am their parent/guardian.

D: …as a caregiver who provides routine care for a child/partner/relative/friend with a NMD

[A response to question 4 is mandatory as this determines the subsequent sequence of questionnaires]

If you are completing this survey on behalf of someone else, please answer all questions as they would answer.

4. What is the month and year of your birth?

5. What was your sex at birth? [Male/Female/Prefer not to answer/Don’t know].

6. What are the first 3 digits in your postal code (Letter Number Letter)?

7. What is your gender?

Male, Female, Transgender Male, Transgender Female, 

Gender Variant/Non-conforming, Non-binary

Not listed, Prefer not to answer, Don’t know

8. To which ethnic or cultural groups did your ancestors belong? Select all that apply.

Canadian, Dutch (Netherlands),  Swedish, French, Chinese,

First Nations (North American Indian), English, Jewish, Métis,

German, Polish, Inuit, Scottish, Portuguese,

Irish, South Asian (e.g., East Indian, Pakistani, Sri Lankan), Italian,

Norwegian, Ukrainian, Welsh, Other (Specify), Don’t Know,

Prefer Not to Answer

9. What languages do you primarily speak at home? Select all that apply.

English, Italian, Polish, French, German, Mandarin/Cantonese, Tagalog (Filipino, Pilipino), Punjabi/Hindi/Urdu, Arabic, Spanish, Portuguese, Other (Specify), Don’t Know, Prefer Not to Answer.

## Appendix 3: NMD classification questionnaire (all participants)

1. How many people in your household are affected by an NMD?

2. (Does not appear for Caregivers without NMD)

What NMD have you been diagnosed with?

Add as many rows as need to specify all their NMD diagnoses. If you do not see their NMD in the suggested list, please enter it in the text box.

3. [Select from List of NMDs in Appendix 4].

4. Specify__________.

5. Age diagnosed _______.

[Click here to add additional diagnoses; Question 2 duplicated each time clicked].

6. (Only appears for Caregivers)

What NMDs have those you care for been diagnosed with?

Add as many rows as need to specify all their NMD diagnoses. If you do not see their NMD in the suggested list, please enter it in the text box.

7. [Select from List of NMDs in Appendix 4].

8. Specify__________.

9. Age diagnosed _______.

[Click here to add additional diagnoses; Question 3 duplicated each time clicked].

## Appendix 4: list of NMDs (all participants)

Myotonic dystrophy type 1 (DM1).

Myotonic dystrophy type 2 (DM2).

Other Myotonic disorder: Specify.

Duchenne muscular dystrophy (DMD).

Becker muscular dystrophy (BMD).

Facioscapulohumeral muscular dystrophy (FSHD).

Oculopharyngeal muscular dystrophy (OPMD).

Limb-girdle muscular dystrophy (LGMD): Specify.

Pompe disease.

Charcot Marie Tooth disease (CMT) neuropathy: Specify.

Other genetic neuropathy: Specify.

Guillain Barre Syndrome (GBS).

Chronic Inflammatory Demyelinating Neuropathy (CIDP).

Other autoimmune neuropathy: Specify.

Congenital myopathies (CM): Specify.

Congenital muscular dystrophy: Specify.

Emery-Dreifuss muscular dystrophy (EDMD).

Other muscular dystrophy: Specify.

Myasthenia gravis.

Lambert-Eaton myasthenic syndrome (LEMS).

Congenital Myasthenic syndrome.

Dermatomyositis.

Necrotizing myopathy HMG/SRP.

Inclusion Body Myositis.

Polymyositis.

Other autoimmune myositis such as antisynthetase syndrome (ASS): Specify.

Distal myopathy or distal muscular dystrophy: Specify.

Spinal muscular atrophy (SMA): Specify.

SMA1

SMA2

SMA3

SMA4

Amyotrophic lateral sclerosis (ALS).

Mitochondrial Disease: Specify.

Kennedy Disease (SBMA).

Friedreich Ataxia.

Autosomal recessive spastic ataxia of Charlevoix-Saguenay (ARSACS).

Metabolic myopathies.

Other neuromuscular disorder: Specify.

Unknown neuromuscular disorder: Specify.

## Appendix 5: equipment costs questionnaire (all participants)

1. In the last five years, how much have you spent on the following equipment or equipment repairs needed for your NMD?

New manual wheelchair $___

Manual wheelchair repair $___

New power wheelchair or scooter $___

Power wheelchair or scooter repair $___

Total other orthopaedic equipment (e.g., leg braces, ankle-foot orthotics, walkers etc.) $___

Non-invasive ventilation devices (e.g., CPAP, BiPAP, CoughAssist etc.) $___

Hospital bed/mattress $___

Other assistive devices/equipment $___ Specify_____________________________________

2. In the last five years, how much have you spent on home modifications needed for your NMD? $___.

3. In the last five years, how much have you spent onvehicle modifications needed for your NMD? $___.

## Appendix 6: health insurance and out-of-pocket costs questionnaire (adults with NMD)

**Questions related to health insurance and out-of-pocket cost**

This section will ask questions related to your incurred costs associated with travel, health insurance, and medication coverage.

**Travel and associated out-of-pocket costs**

This section will gather information about travel costs and other costs associated with your visits to the clinic. Please consider the visits related to your own NMD, and not those under your care.

1. To begin, please confirm: Have you had health care providers (HCPs), such as family physicians and nurses, managing visits for your NMD in the last six months? [Yes/No] [Yes reveals Hidden Questions 2–4].

2. How far have you had to travel to make your HCP managing visits in person? (km).

3. How many visits have you made to your NMD HCP in the last six months?

4. In the last six months, how do you travel to HCP managing visits in person? Select all that apply.

[Car; Public Transit; Taxi/Uber/Lyft; Plane/Train; Other; If Other, Specify_____].

5. What is the total round fare cost for the duration of your trip?

6. What is the average total cost of parking for your NMD HCP managing visits in the last six months? (Please specify the amount in CAD$).

**Costs associated with other health care provider visits**

Please consider the visits related to your own NMD, and not those under your care.

7. How many visits in total have you made for the following type of health care services in the last six months, outside of your typical NMD HCP managing visits? Please answer for those options that apply in the table below.

8. What is the approximate out of pocket cost per visit for other health care services in the last six months, outside of your typical NMD HCP managing visit? Please answer for those options that apply in the table below.

**Table:**

Health care service	Number of visits	Approximate out of pocket cost per visit
Personal Care Attendant/Personal Support Worker		
Massage Therapy		
Physiotherapist/Occupational Therapist		
Chiropractor		
Psychologist/Psychiatrist/Psychotherapist		
Dietitian/Nutritionist		
Respiratory Therapist		
Naturopath/Other Alternative Therapies		
Other		

**Out-of-pocket costs associated with medications**

In this section we will ask about your NMD medications to determine the cost and time burden required to take the prescribed maintenance therapies as recommended by your NMD clinic physicians and staff.

9. I have taken medications in the past six months specifically for my NMD. [Yes/No] [Yes reveals Hidden Question 10]

10. What medications have you taken in the past six months ONLY for your NMD?

**Table:**

Medication	Cost per Month	Covered/Provided by	Specify
		Select: Clinical Trial; Provincial Authorization; Private Insurance Including Insurance through Work; Compassionate Use; Other; Unknown	

[Click here to add another Medication; Adds another row to the table]

11. What type of health insurance for your medication coverage do you have? Select all that apply.

Provincial health insurance plan.

Private health insurance.

12. Did you take any neuromuscular disease medication or related medication during the past six months which was without prescription (not prescribed by a physician and paid for by yourself)? [Yes/No].

Employee/private drug benefit plans could potentially cover 100% of the cost but, many only pay a portion of the costs, and you are therefore left to cover the co-pay as an out-of-pocket (OOP) expense.

If completing on behalf of a person with NMD, please note these questions apply to the person with NMD.

13. What percentage of your medication costs does your plan cover?

[100% / 80% / 70% / 50% / Other (specify)___________ / Prefer Not to Answer / Don’t Know]

## Appendix 7: socioeconomics questionnaire [adults with NMD]

In this section we will ask questions about you and your current employment or schooling circumstances. If you are answering questions on behalf of someone with an NMD, please note that each question relates to the person living with an NMD.

1. What is your marital status?

[Married; Common-law; Single; Widowed; Divorced; Separated; Prefer Not to Answer; Don’t Know]

2. What is the highest level of schooling you completed?

Currently attending secondary/high school.

Some high school.

High school diploma (or equivalent).

Some post-secondary education.

Post-secondary diploma.

Bachelor’s degree or equivalent.

Some Trade/Apprentice School.

Trade/Apprentice School.

Some Graduate School.

Graduate School.

Professional degree (i.e., MD, JD).

3. What is your occupation? Check the box for what best describes your primary occupation. You may check more than option if this applies to your situation.

I am at school, I study.

I am in paid employment or self-employed [Activates Question 4].

I am a homemaker.

I am unemployed.

I am unable to work due to my NMD.

I am retired.

I have taken early retirement due to my NMD.

Other (Specify) _________.

Prefer Not to Answer.

4. If in Paid Employment or Self-Employed, Specify.

Legislative and senior management occupations.

Business, finance, and administration occupations.

Natural and applied science and related occupations.

Health occupations.

Occupations in education, law and social, community and government services.

Occupations in art, culture, recreation, and sport.

Sales and services occupations.

Trades, transport and equipment operators and related occupations.

Natural resources, agriculture and related production occupations.

Occupations in manufacturing and utilities.

Other (Specify) ___________.

Prefer Not to Answer.

Don’t Know.

5. What is your annual pre-tax personal income in the last year?

 < $10,000.

$10,000–20,000.

$20,001–30,000.

$30,001–40,000.

$40,001–50,000.

$50,001–60,000.

$60,001–70,000.

$70,001–80,000.

$80,001—90,000.

$90,001- 100,000.

more than $100,001.

Prefer Not to Answer.

Don’t Know.

6. What is your annual pre-tax household income in the last year?

Less than $5,000

$5,000 to less than $10,000

$10,000 to less than $15,000

$15,000 to less than $20,000

$20,000 to less than $30,000

$30,000 to less than $40,000

$40,000 to less than $50,000

$50,000 to less than $60,000

$60,000 to less than $70,000

$70,000 to less than $80,000

$80,000 to less than $90,000

$90,000 to less than $100,000

$100,000 to less than $150,000

$150,000 to less than $200,000

$200,000 to less than $250,000

$250,000 and over

Prefer Not to Answer

Don’t Know

Thinking about living with NMD, have you as someone living with an NMD had to:

7. Take money from your savings, retirement for your NMD care/support? [Yes/No].

8. Relocate to be closer to NMD care, or treatment/support? [Yes/No].

9. Skip a medication or treatment because you could not afford the medicine? [Yes/No].

10. Skip a clinic visit or doctor’s appointment because you could not afford to miss time off work/school? [Yes/No].

11. Received Disability Tax Credit (DTC)? [Yes/No].

## Appendix 8: caregiver supports & impacts questionnaire

The following set of questions is for those who are currently caring for a person with an NMD.

In this section, we ask questions about time spent away from work or school due to caring for the person with an NMD. Please note these questions apply to you as a caregiver and not the person with the NMD.

1. Thinking about living with an NMD, have you as a caregiver for someone with an NMD had to relocate to be closer to their place of NMD care, or treatment/support? [Yes/No].

2. What kinds of support would you like to have? Select all that apply.

Home care or support

Financial support, government assistance or tax credit

Information or advice

Emotional support or counselling

Help from medical professionals

Occasional relief or respite care

Volunteer services or community services

Other (Specify)

3. Is there any other type of support that you would like to have to help with your caregiving duties?

4. There are many ways of handling difficult situations. In the last six months, have you used any specific coping methods to help you deal with your caregiving responsibilities? Select all that apply.

Exercising, walking or yoga

Professional counselling or therapy

Socializing or talking to friends or other caregivers

Religious or spiritual practices, or meditation

Reading, watching television or listening to music

Other (Specify)

5. During the last six months, have you had any out-of-pocket expenses because of your caregiving responsibilities? These costs are specific to the caregiver and include any costs that are different from costs required by the person with NMD. [Yes/No].

If yes, how much did you spend?

Less than $200

$200 to <$500

$500 to <$1000

$1000 to <$2000

$2000 to <$5000

$5000 or more

6. During the last six months, have you experienced financial hardship because of your caregiving responsibilities? [Yes/No].

7. During the last six months have you had to: Select all that apply.

Borrow money from family or friends

Take loans from a bank or financial institution

Use or defer savings

Modify your spending

Sell off assets

File for bankruptcy

Other (Specify)

## Appendix 9: productivity measures questionnaire

We will now ask you questions regarding your current work status and the time and productivity that you have lost as a result of your NMD.

f you are answering questions on behalf of someone that has an NMD, please note that each question relates to the person living with an NMD.

1. In the last six months, were/are you employed or attending school? (Select one).

I was/am employed, but not attending school [Reveals Hidden Questions 3, 5, 6, 7, 8]

I was/am attending school, but not employed [Reveals Hidden Questions 4, 5, 6, 7, 8]

I was/am employed and attending school [Reveals Hidden Questions 3, 5, 6, 7, 8]

I was/am not employed nor attending school [Reveals Hidden Question 2]

2. If not working or attending school, is it due to your NMD? [Yes/No].

3. Have you been absent from your work or school in the last six months because of your neuromuscular condition? [Yes/No] [Yes reveals Hidden Question 4].

4. During the last six months, how many days did you miss from work or school because of your NMD? (Only count the working/school days in the last 6 months. Include the days missed for sick days and the days you left work or school early.)

5. During the last six months, how many days did you miss from work or school because of any other reason, such as vacation, holidays, time off for any other reason? (Only count working/school days).

6. During the last six months, how many days did you actually work or attend school? (Only count working/school days).

7. On the days when you are at work or school, perhaps you were not able to do as much work as normal due to your NMD. On those days, how much work could you do on average? [VAS 0–10].

0 – I was not able to do anything those days

5- I could do around half

10 – I was able to do just as much as normal

8. During the last six months, how much did your NMD affect your productivity while you were either working or at school? Think about the days you were limited in the amount or kind of work or schooling you expect of yourself, or the days you could not do these as carefully as your usual. If your NMD affected your work or schooling only a little, choose a low number. Choose a high number if your NMD affected your work or school a great deal. Consider only how much health problems affected productivity while you were working. [VAS 0–10].

0 – Health problems had no effect on my work or schooling

10 – Health problems completely prevented me from working or schooling

9. During the last six months, how much did your NMD affect your ability to do your regular daily activities, those activities other than your work or schooling? By regular daily activities, we mean the usual activities you do such as work around the house, shopping, childcare, exercising, studying, etc. Think about times you were limited in the amount or kind of activities you could usually do and times you accomplished less than you would like. If your NMD affected your activities only a little, then choose a low number. Choose a high number if your NMD affected your activities a great deal. Consider only how much health problems affected your ability to do your regular daily activities, other than work at a job. [VAS 0–10].

0 – Health problems had no effect on my regular daily activities

10 - Health problems completely prevented me from doing regular daily activities

## Appendix 10: school impact questionnaire (minors with NMD only)

Please note that the following questions are related to the child living with an NMD. Please answer on his/her behalf to the best of your ability.

1. Is your child currently enrolled in school or pre-school?

[Yes/No/Prefer Not to Answer/Don’t Know]

2. If your child has attended school, what is the highest level they achieved?

Currently pre-school aged

Currently attending elementary/primary school

Currently attending secondary/high school

Some high school

High school diploma (or equivalent)

Some post-secondary education

Post-secondary diploma

Bachelor’s degree or equivalent

Some Trade/Apprentice School

Trade/Apprentice Certificate

3. Has your child's NMD caused them to miss any school in the past 7 days?

[Yes/No/Prefer Not to Answer/Don’t Know]

4. Thinking about your child's whole life, how much school have they missed because of their NMD?

[None/A Little Bit/A Fair Amount/A Lot/All of It/Prefer Not to Answer/Don’t Know]

## Appendix 11: survey conclusion (all participants)

Do you need to complete this survey again for another household member with an NMD or as a Caregiver for a person with an NMD?Yes/No.

You will be provided with a link to re-start the survey on the next page. You can click it to start the next survey right away, or save it to use later. It contains a special code so all the responses from a single household can be linked.

[Click Here to End Survey].

…

…

…

You have reached the end of the survey!

Thank you for your participation, we appreciate the time you took to answer all questions in the questionnaires!

Link to complete this survey again for another household member with an NMD or as a Caregiver for a person with an NMD *[link to restart a new survey with a household linkage ID].*

Link to the Muscular Dystrophy Canada website https://muscle.ca/.

If you have any questions, please don’t hesitate to contact the Clinical Research Associate, *[Name]*, via email at *[Email Address]*.

## Abbreviations

BIND: Burden of Inherited Neuromuscular Diseases
CIIQ: Caregiver Indirect and Informal Care Cost Assessment Questionnaire
CNDR: Canadian Neuromuscular Disease Registry
HRQoL: Health-related Quality of Life
HUI: Health Utilities Index
MDC: Muscular Dystrophy Canada
NMD: Neuromuscular Disorder
PedsQL: Pediatric Quality of Life Inventory
QALE: Quality-adjusted Life Expectancy
SGBA: Sex and Gender Based Analysis
WPAI: Work Productivity and Activity Impairment questionnaire
